# IMPACT OF THE COVID-19 PANDEMIC ON FRONTLINE PAID DEMENTIA WORKFORCE STAFF

**DOI:** 10.1093/geroni/igad104.1770

**Published:** 2023-12-21

**Authors:** Esteban Barreto

**Affiliations:** Massachusetts General Hospital, Boston, Massachusetts, United States

## Abstract

**Background:**

Patients, families and the frontline paid workforce caring for persons with dementia faced dramatic change and high risk in the time of COVID-19 due to underlying vulnerability toward the disease as well as isolation, lockdown, and visitation restrictions. Purpose: The purpose of this study was to examine the paid and informal care workforce caring for persons with dementia at home, in assisted living and skilled nursing facilities. This analysis will focus on the impact on paid workers in ALF and SNF.

**Methods:**

We conducted online and paper surveys of 182 workers caring for persons with dementia during the COVID-19 pandemic in several assisted living facilities in New England (n=82) and a large skilled nursing facility in Florida (n=100)

**Results:**

A majority of workers were not married and had less than college level education. 66% knew patients who died of COVID-19; 90% or more said they were exposed to the virus personally. Half reported mild, moderate or severe changes due to the pandemic on their personal access to health care, food security and sleep. Workers in ALF were more likely to report only good or fair personal health status. Most workers indicated they had sufficient education about infection control.

**Conclusion:**

The workforce in long term services and supports has been the slowest to recover from job losses during the pandemic. Further studies are needed to inform the health and social needs of workers caring for persons with dementia.

